# The connecting health and technology study: a 6-month randomized controlled trial to improve nutrition behaviours using a mobile food record and text messaging support in young adults

**DOI:** 10.1186/s12966-016-0376-8

**Published:** 2016-04-21

**Authors:** Deborah A. Kerr, Amelia J. Harray, Christina M. Pollard, Satvinder S. Dhaliwal, Edward J. Delp, Peter A. Howat, Mark R. Pickering, Ziad Ahmad, Xingqiong Meng, Iain S. Pratt, Janine L. Wright, Katherine R. Kerr, Carol J. Boushey

**Affiliations:** School of Public Health, Curtin University, GPO Box U1987, Bentley, Perth, WA 6845 Australia; Public Health Division, Department of Health In Western Australia, 189 Royal Street, East Perth, 6004 WA Australia; Video and Image Processing Laboratory, School of Electrical and Computer Engineering, Purdue University, West Lafayette, IN USA; School of Engineering and Information Technology, The University of New South Wales at the Australian Defence Force Academy, Canberra, Australia; School of Medicine, Flinders University, Bedford Park, Australia; Cancer Council Western Australia, Subiaco, WA Australia; Epidemiology Program, University of Hawaii Cancer Center, Honolulu, HI USA; Department of Nutrition Science, Purdue University, West Lafayette, IN USA

**Keywords:** Mobile food record, Novel technology, Dietary assessment, Interventions, Text messaging, Young adult, Tailoring, Energy-dense nutrient poor foods, Sugar-sweetened beverages, Fruit, Vegetables, Junk food

## Abstract

**Background:**

Early adulthood represents the transition to independent living which is a period when changes in diet and body weight are likely to occur. This presents an ideal time for health interventions to reduce the effect of health problems and risk factors for chronic disease in later life. As young adults are high users of mobile devices, interventions that use this technology may improve engagement. The Connecting Health and Technology study aimed to evaluate the effectiveness of tailored dietary feedback and weekly text messaging to improve dietary intake of fruit, vegetables and junk food over 6 months among a population-based sample of men and women (aged 18–30 years).

**Methods:**

A three-arm, parallel, randomized control trial was conducted. After baseline assessments, participants were randomized to one of three groups: A) dietary feedback and weekly text messages, B) dietary feedback only or C) control group. Dietary intake was assessed using a mobile food record App (mFR) where participants captured images of foods and beverages consumed over 4-days at baseline and post-intervention. The primary outcomes were changes in serves of fruits, vegetables, energy-dense nutrient-poor (EDNP) foods and sugar-sweetened beverages (SSB). The intervention effects were assessed using linear mixed effect models for change in food group serves.

**Results:**

Young adults (*n* = 247) were randomized to group A (*n* = 82), group B (*n* = 83), or group C (*n* = 82). Overall, no changes in food group serves for either intervention groups were observed. An unanticipated outcome was a mean weight reduction of 1.7 kg (*P = .02*) among the dietary feedback only. Men who received dietary feedback only, significantly reduced their serves of EDNP foods by a mean of 1.4 serves/day (*P = .02*). Women who received dietary feedback only significantly reduced their intake of SSB (*P = .04*) by an average of 0.2 serves/day compared with controls.

**Conclusions:**

Tailored dietary feedback only resulted in a decrease in EDNP foods in men and SSB in women, together with a reduction in body weight. Using a mobile food record for dietary assessment and tailored feedback has great potential for future health promotion interventions targeting diet and weight in young adults.

**Trial Registration:**

Australian Clinical Trials Registry Registration number: ACTRN12612000250831.

## Background

There is convincing evidence of the importance of regularly eating a healthful diet for the prevention of chronic diseases and excessive weight gain in adulthood, particularly a diet high in fruits and vegetables and that limits energy-dense nutrient-poor (EDNP) foods and beverages [1]. Chronic diseases, such as obesity, cardiovascular disease and some cancers are diet related [2] and interventions targeting early adulthood may reduce the effect of health problems and risk factors for chronic disease in later life. In 2011, over half of young adults aged 18–24 years and 59 % of 25–34 year olds in Western Australia were classified as either overweight or obese [3]. Weight gain in early adulthood has been attributed to less physical activity and excess energy intake as well as the obesogenic environment [4]. In Australia, teenagers and young adults consume more energy dense nutrient poor foods (EDNP) such as fast food, chocolate, chips, meat pies, pizzas and sugar-sweetened beverages (SSB) than other age groups and are less likely than older adults to meet the Australian guidelines of at least two 150 g serves of fruit and five 75 g serves of vegetables a day [5]. These statistics may result from the challenges of early adulthood being a time of transitioning to independent living and starting a family.

In a systematic review of lifestyle interventions for preventing weight gain in young adults, Hebden et al. [6] recommended future trials include dietary self-monitoring and tailored feedback to increase the personal relevance to the individual. Dietary self-monitoring is commonly undertaken as a written food record by asking the person to record the types of amounts of all foods and beverages consumed over one or more days. The act of recording appears to raise a person’s awareness of what they are eating and has been shown to be an effective behaviour change strategy [7]. However, many weight loss studies where food records have been used for self-monitoring fail to include sufficient detail for assessment of diet to be undertaken or measures of adherence, such as the day and time of recording [7]. Food records, also referred to as food diaries, can provide an assessment of overall dietary intake, including details of the foods consumed and food combinations eaten together [8] but tend to be less acceptable in young people due to the recording burden [9]. With mobile technology being more readily accessible, digital and image-based diet assessment methods may address some of these limitations, allowing for simultaneous dietary assessment and self-monitoring. Given the level of interest in mobile technology amongst young adults, collecting dietary intake data using mobile devices may have more appeal and lead to improved cooperation in this age group. An additional advantage is the detailed information collected can form the basis of tailored dietary feedback for the individual. Tailoring is a form of communication personalised to the individual based on characteristics unique to that person and derived from individual assessment [10]. A key element of successful tailoring is to provide personally relevant feedback that can assist people to identify the dietary changes most likely to improve their health [11]. Tailoring has shown positive effects in changing diet and physical activity behaviours [12, 13]. Tailored dietary feedback has been delivered by mail and web but to date, text messaging as a mode of delivery for feedback has been relatively unexplored. Most studies have based their tailored feedback on brief instruments that use only a few questions to assess diet rather than more detailed dietary records [13]. A systematic review of dietary assessment methods used to evaluate interventions found that dietary components, such as fruits, vegetables, sugar-sweetened beverages and fast food, were most often assessed by single questions or brief instruments [14]. This limits the type and quality of feedback that can be provided to the participant. However, more detailed methods such as paper-based food records can be more burdensome for the participant leading to poorer acceptability.

In response to these concerns with dietary assessment, the investigators have developed an image-based dietary assessment system known as Technology Assisted Dietary Assessment or TADA [15, 16]. The mobile food record (mFR) App uses a camera to capture before and after images of food and beverages consumed. The Connecting Health and Technology (CHAT) study was the first intervention study to assess diet with the mFR and provide tailored dietary feedback with text messaging support to engage participants in making dietary changes. The CHAT study was undertaken as a 6-month randomized control trial (RCT) among young adults to investigate the effectiveness of tailored feedback and weekly text messaging as a method to increase serves of fruits and vegetables and decrease serves of EDNP food and SSB compared with a group receiving only tailored dietary feedback and a control group who did not receive any dietary feedback or text messages.

## Methods

### Design

**Fig. 1:**
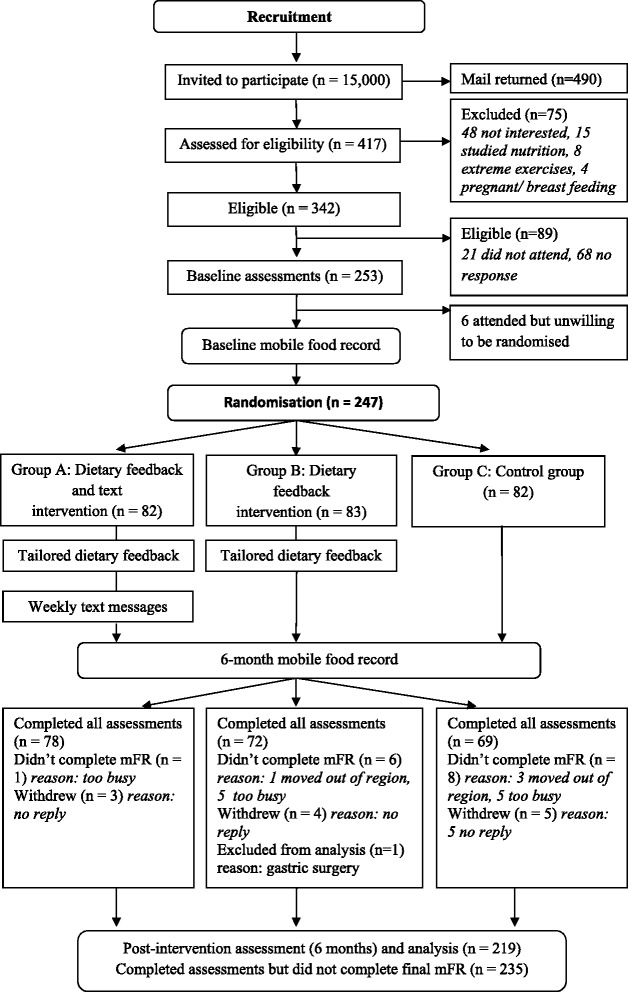
Participant flow diagram

The study was a 6-month RCT to evaluate the effectiveness of a tailored dietary feedback and text messaging support in young adults aged 18 to 30 years (Fig. 1). The trial was registered (Australian Clinical Trials Registry Registration number ACTRN12612000250831) and the protocol published [17]. The tailored intervention was based on self-determination theory (SDT) and informed by motivational interviewing (MI) [18–20]. The project was referred to as the Connecting Health and Technology (CHAT) study. The project protocol was approved by the Curtin University Human Research Ethics Committee and the Department of Health, Western Australia Human Research Ethics Committee and all participants signed an informed consent.

### Participants

Young adults aged 18–30 years were recruited from the Federal Electoral Roll, a compulsory enrolment system for Australians aged over 18 years. They were selected from 57 suburbs within the Perth metropolitan area to provide representation across socio-economic status [21]. A printed letter of invitation was mailed out by an independent company, as the researchers were not permitted direct access to the mailing list. After receiving the letter of invitation, those who wished to take part in the study contacted the research team by email, mobile telephone (text or voice), landline telephone or the study website. Other recruitment methods supplemented the mail out and included advertising on the University website, flyers posted on campus and referrals from friends or colleagues. The majority of participants were recruited through the electoral roll mail out (approximately 73 %).

Participants were screened for eligibility by completing a web form or by telephone and were aged between 18 and 30 years as of their last birthday and owned a mobile telephone. Exclusion criteria applied if people were unable to complete the 6 month study, undertaking extreme forms of exercise (for example, marathon training) or on a special diet (for example, strict weight loss diet or following a restrictive diet that excluded food groups), currently studying or had studied nutrition, pregnant or breastfeeding, unable to attend the study centre to complete the face-to-face assessments or if they had any serious illnesses. A participant flow diagram (Fig. 1) outlines the reasons for exclusion.

### Data collection

**Fig. 2:**
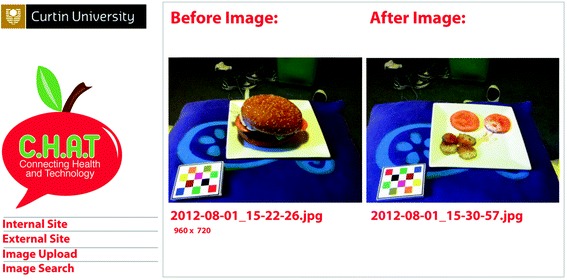
View of the website with before and after images of an eating occasion and metadata from the mobile food record images

Participants who met the selection criteria were invited to attend two face-to-face baseline data collection visits one week apart. At the first visit they had their height and weight measured, completed written questionnaires and underwent training on how to use the mFR App for the collection of dietary information. One of three research staff conducted each training session on how to: connect to Wi-Fi for sending images; take a practice image of plastic food replicas; and send the before and after image pair to the back-end server. Participants were instructed to record their food and beverage intake using the mFR for four consecutive days (Wednesday to Saturday) with the investigator-supplied iPod Touch (iOS6) loaded with the mFR App. When taking an image, participants were instructed to include a reference device known as a fiducial marker (shown in Fig. 2) to assist with food identification and portion size estimation. They were instructed to record food and beverage items not captured using the iPod notes section or in a small booklet provided.

The mFR App had an automated feature to detect the presence of the fiducial marker and alerted participants if the fiducial marker was missing from the image. An angle-detection algorithm assisted participants to take the image at the correct angle by a light turning green when the angle of the mobile device was positioned between 45 and 60° from the horizontal plane. Once captured, the images were not accessible to the participant. The mFR App and the back-end server were adapted for use in this project [16, 17]. In the current study, the trained analyst confirmed the contents of the images and probed for any forgotten recordings with participants. Previous work with the mFR, showed no difference between the reported energy intake and estimated energy requirements [22]. The back-end server was password protected and images were stored with a unique password protected participant ID that was entered into the mFR App by the researcher. The App performed automatic uploading of food and beverage images collected by participants when in Wi-Fi range. If participants did not have access to Wi-Fi, their images were stored securely in the App until a Wi-Fi connection was made.

A week later participants attended a second baseline visit to return the iPod Touch and complete additional written questionnaires. At this visit the research dietitian interviewed each participant to verify the content of the images and probe for any forgotten food and beverages. A computer software generated randomisation table was then used to assign each participant to one of three treatment groups 1) combined dietary feedback and weekly text messages, 2) dietary feedback or 3) control group. Sequence generation was conducted by a biostatistician not involved in the implementation of the trial on site, and therefore was not in contact with the study participants. The control group recorded their dietary intake using the mFR at baseline and again at 6 months completion but did not receive dietary feedback until the end of the study. At six months, all participants completed questionnaires, the 4-day mFR and had their weight measured. All participants received a $20 gift voucher of their choice at baseline and six months and were entered into a prize draw to win an iPad, iPod or shopping voucher at the end of the study.

### Dietary analysis

A trained analyst (a research dietitian) viewed the before and after images simultaneously for food identification and estimation of amount eaten. When needed the trained analyst clarified with participants the contents of the images and checked for any forgotten food or beverages not reported. The trained analyst assessed the 4-day mFRs using a quality scoring of food items by food group (serves of fruits, vegetables and EDNP food and beverages according to the Australian Guide to Healthy Eating standard serves (AGHE) [23]. AGHE serving sizes specify one serve of fruit is equivalent to 150 g, one serve of vegetables is equivalent to 75 g, and one serve of EDNP foods or beverages is equivalent to the amount of approximately 600 kilojoules (143 kilocalories). Note that the AGHE includes fried potato as an EDNP food not a vegetable serve. A purpose-built Microsoft Access data table was developed for food and beverages data entry with linked categories for food group, food type and serving size. The same trained analyst entered all data from the mobile food record for both the baseline and final visit. The time taken to enter each 4-day record varied between 20 and 30 min. To assist with portion size estimation the trained analyst used the fiducial marker in the image served as a reference for size. For each participant, an average serve per day was calculated for fruits, vegetables, SSB, EDNP foods and alcohol.

### Dietary feedback messages

**Fig. 3:**
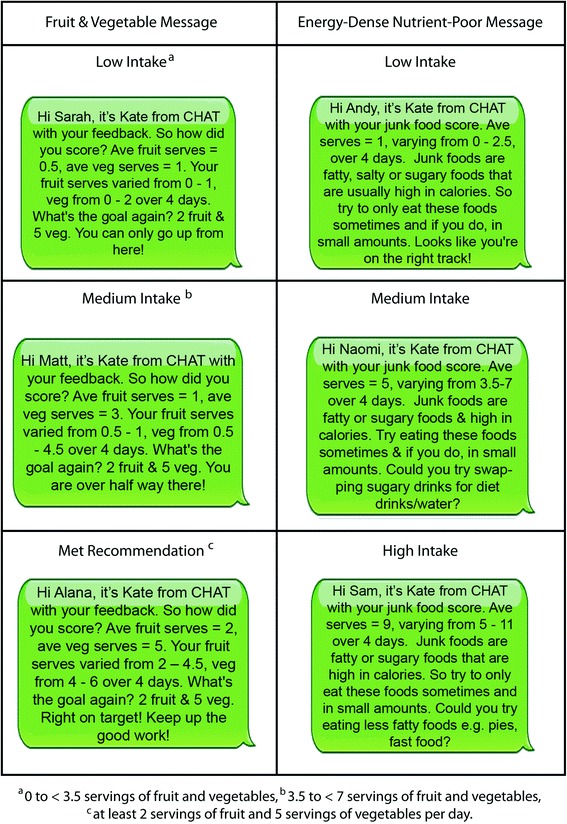
Examples of the tailored dietary feedback text messages on fruits and vegetables and energy-dense nutrient-poor foods, for the intervention arms: dietary feedback and text messaging; dietary feedback only

Once the scoring was complete, two tailored dietary feedback text messages (Fig. 3) were constructed for the intervention participants, i.e., the dietary feedback and weekly text messages group and the dietary feedback only group, with one message for fruits and vegetables and the other for EDNP food and SSB. A standard message template was used for each dietary feedback text message but modified for each participant according to the results of the dietary analysis (Fig. 3). For the fruit and vegetable message, a scripted message was devised for three levels of intake: (1) low: 0 to < 3.5 servings of fruits and vegetables; (2) medium: 3.5 to < 7 servings of fruits and vegetables; and (3) met recommendation: at least 2 servings of fruits and 5 servings of vegetables per day. For EDNP serves, a library of messages was developed and modified according to the participant’s dietary intake for EDNP serves. For example, “…could you try eating less sugary foods?”; “could you try eating less fast food or takeaway foods?”. As there is no recommended servings for EDNP foods and beverages, 0–3 serves were considered a low intake and the message included the text “looks like you are on the right track”. At EDNP serves of 3 or more per day, the message was personalised with key sources of EDNP serves identified from the mobile food record. For example, the message in Fig. 3: “could you try swapping sugary drinks for diet drinks/water?” indicates that for this individual sugary drinks were a key source of EDNP serves. In developing the text messages several tailoring strategies were used. The message was personalised with the individual’s name and the feedback strategy was descriptive and evaluative [24]. The language and tone of voice of the dietary feedback messages were based on results of message preference testing with focus groups [25] and designed to be an autonomous supportive style of communication [26]. The two text messages were sent one week apart, using an automatic text message delivery system. Alcohol intake was not addressed in the message as this was not the target behaviour for the intervention.

### Weekly text messages

The group receiving dietary feedback and weekly text messages, were sent text messages to their mobile telephone for six months. The motivational and informative messages focused on fruits, vegetables and junk foods and beverages. The text message content was based on formative focus group work testing the potential persuasiveness of messages for use in the intervention [25]. We used an autonomous supportive style of communication (pull vs push messages) and avoided offering direct advice consistent with motivational interviewing principles [26]. Offering substitutes and using an empathetic tone guided message construction. For example, “Running late, no time to make lunch, so you end up eating junk? How about a soup or sandwich - it's quick and healthy too!” or “Isn’t it easy to reach for unhealthy snacks when you’re hungry? So maybe keep some fruit handy for when those hunger pangs hit!”. The message also included web links to recipes and nutrition information. The Go for 2&5® campaign recipes [27], developed and tested against nutrition criteria to meet the AGHE, were adapted for readability and suitability for smartphone viewing. A total of 32 messages were sent once or twice a week over a 24 week period. They were delivered between four and six pm on different days of the week to minimise their predictability. Participants were able to stop receiving text messages at any point by replying “stop”.

### Outcome measures

The primary outcome variables measured at baseline and post intervention, were the serves of fruits, vegetables, SSB and EDNP foods consumed each day. Height and weight were measured according to a standard protocol [28]. Demographic and personal characteristics (sex, age, eating behaviour, educational level, country of birth, ethnicity, living arrangements, socioeconomic status, financial status, cooking abilities, attitudes towards eating a healthy diet, perception of their body weight, intake of fruits, vegetables, junk and alcohol intake and recent dietary changes) were assessed using written questionnaires [29]. Physical activity was assessed using the International Physical Activity Questionnaire (IPAQ) Short Form and the results reported as MET- minutes per week according to the recommended method of scoring [30]. Based on a motivational interviewing strategy, importance, confidence and motivation to change behaviour with regards to the primary outcomes were examined using a 10 point rating scale; for example, ‘How important is eating a healthy diet to you? One was not at all important and 10 was very important’ [26]. The responses to these questions were categorized as low for scores 0-5 and high for scores 6–10.

### Statistical analysis

The primary outcomes were changes in servings of fruit, vegetables, SSB and EDNP foods in the three groups (dietary feedback and text messaging; dietary feedback; and control group). Changes from baseline to 6 months were assessed using the paired-sample *t* test. Secondary outcomes were changes in body weight and BMI. The intervention effects (dietary feedback and text message compared to control and dietary feedback only compared to control) at 6 months were assessed using linear mixed effect models for continuous variables (change in serves). Differences between treatment groups are expressed as mean change in serves and associated 95 % confidence interval (CI). Logistic regression analyses were used to assess whether there were differences between groups in a change of 0.5 serves in targeted foods and odds ratio along with 95 % CI. Data were analysed using Stata MP 14.0 (Texas, US) and *P* values < 0.05 (2 tailed) were considered as statistically significant.

## Results

Table 1 shows the participant characteristics at baseline according to the study group. The data shows an even distribution across age, BMI category, ethnicity, education level, alcohol and smoking status. At baseline, there were no significant differences in the intake of food groups between the three study groups. In total, 220 of the 247 participants completed the intervention, which resulted in an 89 % retention rate at six months. Figure 1 shows the reasons for non-completion. The final sample was 219 as one participant underwent gastric surgery for obesity and was excluded from the analysis. A further 15 participants who were unable to undertake the final mFR completed on-line questionaries. Two dietary feedback and text messaging participants elected to stop receiving messages.

**Table 1:** Characteristics of study participants randomised at baseline (*n* = 247) comparing dietary feedback and text messages, dietary feedback only and control group

	Feedback + Text (*n* = 82)	Feedback only (*n* = 83)	Control (*n* = 82)
Men	29	28	28
Women	53	55	54
Mean ± SD			
Age (years)	24.2 ± 3.2	23.7 ± 3.4	25.0 ± 3.5
Height (cm)	168.8 ± 10.1	168.9 ± 9.1	170.9 ± 8.8
Weight (kg)	67.9 ± 14.1	70.4 ± 17.7	71.9 ± 17.6
Body Mass Index (kg/m^2^)	23.8 ± 4.1	24.7 ± 6.2	24.6 ± 5.6
BMI category (%)			
Underweight < 18.5 (kg/m^2^)	11.0	12.0	4.9
Healthy weight 18 .5–24.9 (kg/m^2^)	58.5	50.6	65.9
Overweight 25–29.9 (kg/m^2^)	20.7	25.3	13.4
Obese ≥ 30(kg/m^2^)	9.8	12.0	15.9
Ethnicity (%)			
White	76.8	77.1	78.0
Aboriginal	0.0	1.2	3.7
Asian	23.2	12.0	14.6
Pacific Islander	0.0	0.0	0.0
Black	0.0	1.2	0.0
Mixed race	0.0	7.2	3.7
Level of Education (%)			
Year 12 or lower	31.7	41.0	35.4
Trade or diploma	25.6	27.7	19.5
Bachelor degree or higher	42.7	31.3	45.1
Alcohol status (%)			
Never drink alcohol	14.8	14.5	8.5
1–4 times a month	59.3	54.2	62.2
2 or more times a week	25.9	31.3	29.3
Smoking status (%)			
Never smoked	65.4	69.9	70.7
Former smoker	28.4	26.5	23.2
Current smoker	6.2	3.6	6.1
Physical Activity mean ± SD			
Total MET minutes per week	2814 ± 2876	2926 ± 3073	3155 ± 2844
Importance of eating a healthy diet mean ± SD^b^			
Score	7.6 ± 1.6	7.3 ± 1.6	7.8 ± 1.5
Food group servings (mean daily serves ± SD)^a^			
Fruit serves (150 g)	1.1 ± 1.1	1.0 ± 1.1	0.9 ± 0.8
Vegetable serves (75 g)	2.0 ± 1.0	1.7 ± 0.9	1.9 ± 1.1
EDNP food serves	3.1 ± 1.5	3.3 ± 1.8	3.1 ± 1.7
EDNP (sugar-sweetened) beverages	0.5 ± 0.6	0.5 ± 0.7	0.4 ± 0.5
Alcohol serves	0.6 ± 0.8	0.7 ± 1.3	0.5 ± 0.7
Total EDNP food & beverages	4.2 ± 1.9	4.5 ± 2.7	4.0 ± 2.1

^a^Serving sizes based on Australian Guide to Health Eating (AGHE). EDNP serves ~ 600 kilojoules equivalents

^b^Question was ‘How important is eating a healthy diet to you?’used a 10 point rating from zero ‘not at all important’ to 10 ‘very important’

Forty-seven percent of the participants were employed full-time and 20 % were students, 37 % lived with their parents and 27 % lived with a partner (with no children) and 16 % lived with friends. Forty-one percent of participants shared some responsibility for the household’s food shopping, 33 % were the main food shopper and 23 % had little or no responsibility. The majority of participants (46 %) had shared food preparation responsibility, 30 % were the main food preparer and 19 % had little or no food preparation responsibility. Most participants said they could cook, 69 % were able to cook a wide variety of meals or almost anything, 25 % reported they could prepare a ‘basic meat and three veg’ meal, whilst 5 % reported being able to boil an egg, barbecue or heat frozen meals.

Each participant used a study provided iPod. As for the study participants’ own mobile telephone ownership, there were only six participants (2.4 %) whose mobile telephone did not have smartphone capabilities. Approximately 56 % of participants owned an iPhone and 25 % owned an Android smartphone.

The effects of the intervention within each study arm and between group differences for the outcome variables are shown in Table 2. No significant differences were observed in food group serves for the group receiving dietary feedback and weekly text messages or for the group receiving only dietary feedback. Compared to baseline, at the end of the 6-months study, the dietary feedback and weekly text intervention group significantly reduced EDNP food. The dietary feedback only intervention arm increased vegetable intake and reduced sugar-sweetened beverage and EDNP food, and the control group significantly increased vegetable intake (Table 2). Subgroup analysis by gender did appear to show a different response to the intervention. Men who received dietary feedback only, significantly reduced their EDNP foods compared with controls (*P = .02*). For women in the dietary feedback only group compared to the control group, there was a significant reduction in SSB serves (*P = .04*) compared to the control group. Compared to baseline, women in all three groups significantly increased their vegetables serves (Table 2 all *P < 0.05)* and reduced their EDNP foods (Table 2 all *P < .05*).

**Table 2:** The change in food groups serves per day, body weight and BMI within trial groups

	^a^Mean ± SEM (6 months – baseline)			^b^Between group difference in Mean change [95 % CI]	
	Feedback + Text (*n* = 78)	Feedback only (*n* = 72)	Control (*n* = 69)	Feedback + Text-Control	Feedback only-Control
All participants					
Vegetables serves	0.2 ± 0.1	**0.4 ± 0.1** ***P = .002***	**0.4 ± 0.1** ***P = .02***	−0.1 [−0.5,0.2]	0.1 [−0.3,0.4]
Fruit serves	**−0.2 ± 0.1** ***P = .03***	−0.1 ± 0.1	−0.2 ± 0.1	−0.1 [−0.4,0.2]	0.1 [−0.2,0.4]
Sugar-sweetened beverage serves	−0.1 ± 0.1	**−0.2 ± 0.1** ***P = .02***	−0.1 ± 0.1	0.1 [−0.2,0.3]	−0.1 [−0.3,0.1]
EDNP food serves	**−0.8 ± 0.2** ***P < 0.001***	**−0.8 ± 0.2** ***P < 0.001***	−0.5 ± 0.2	−0.3 [−0.9,0.3]	−0.4 [1.0,0.2]
Alcohol serves	−0.1 ± 0.1	−0.2 ± 0.1	0.0 ± 0.1	−0.1 [−0.4,0.2]	−0.1 [−0.4,0.2]
Body weight (kg)	0.4 ± 0.4	−0.6 ± 0.5	1.1 ± 0.7	−0.8 [−2.2,0.7]	**−1.7 [−3.2,−0.3]** ***P = .02***
BMI	0.1 ± 0.1	−0.3 ± 0.2	0.4 ± 0.2	−0.2 [−0.7,0.3]	**−0.6 [−1.1,−0.1]** ***P = .02***
Men					
Vegetables serves	−0.2 ± 0.2	0.2 ± 0.3	0.2 ± 0.2	−0.4 [−0.9,0.2]	0.0 [−0.6,0.6]
Fruit serves	−0.5 ± 0.3	−0.2 ± 0.3	−0.3 ± 0.2	−0.3 [−0.9,0.4]	0.0 [−0.7,0.7]
Sugar-sweetened beverage serves	0.0 ± 0.2	0.0 ± 0.2	−0.1 ± 0.1	0.2 [−0.3,0.6]	0.2 [−0.3,0.7]
EDNP food serves	**−1.0 ± 0.4** ***P = .01***	**−1.4 ± 0.5** ***P = .008***	−0.0 ± 0.4	−0.9 [−2.1,0.3]	**−1.4 [−2.6,−0.2]** ***P = .02***
Alcohol serves	−0.1 ± 0.1	0.0 ± 0.3	0.0 ± 0.1	−0.1 [−0.6,0.5]	−0.1 [−0.5,0.6]
Body weight (kg)	0.6 ± 0.7	0.3 ± 0.8	1.8 ± 1.9	−1.3 [−4.4,1.9]	−1.5 [−4.8,1.8]
BMI	0.2 ± 0.2	0.1 ± 0.2	0.6 ± 0.6	−0.4 [−1.4,0.6]	−0.5 [−1.5,0.5]
Women					
Vegetables serves	**0.4 ± 0.2** ***P = .01***	**0.5 ± 0.1** ***P < .001***	**0.4 ± 0.2** ***P = .03***	−0.2 [−.05,0.4]	0.1 [−0.4,0.6]
Fruit serves	−0.2 ± 0.1	0.0 ± 0.1	−0.2 ± 0.1	0.0 [−0.3,0.3]	0.1 [−0.2,0.4]
Sugar-sweetened beverage serves	−0.1 ± 0.1	**−0.3 ± 0.1** ***P = .001***	−0.1 ± 0.1	0.0 [−0.2,0.3]	**−0.2 [−0.4,−0.01]** ***P = .04***
EDNP food serves	**−0.7 ± 0.2** ***P = .001***	**−0.5 ± 0.2** ***P = .03***	**−0.6 ± 0.3** ***P = .02***	0.0 [−0.7,0.6]	0.1 [−0.5,0.8]
Alcohol serves	−0.3 ± 0.1	−0.3 ± 0.1	0.0 ± 0.1	−0.1 [−0.4,0.2]	−0.2 [−0.5,0.1]
Body weight (kg)	0.3 ± 0.5	−1.0 ± 0.6	0.8 ± 0.5	−0.5 [−2.0,0.9]	**−1.8 [−3.3,−0.4]** ***P = .01***
BMI	0.1 ± 0.2	−0.4 ± 0.2	0.3 ± 0.2	−0.2 [−0.7,0.4]	**−0.7 [−1.3,−0.2]** ***P = .01***

^a^Paired-sample *t* test was used to assess within group differences

^b^Linear mixed models was used to assess between group differences

Bold values denote significant treatment differences

**Fig. 4:**
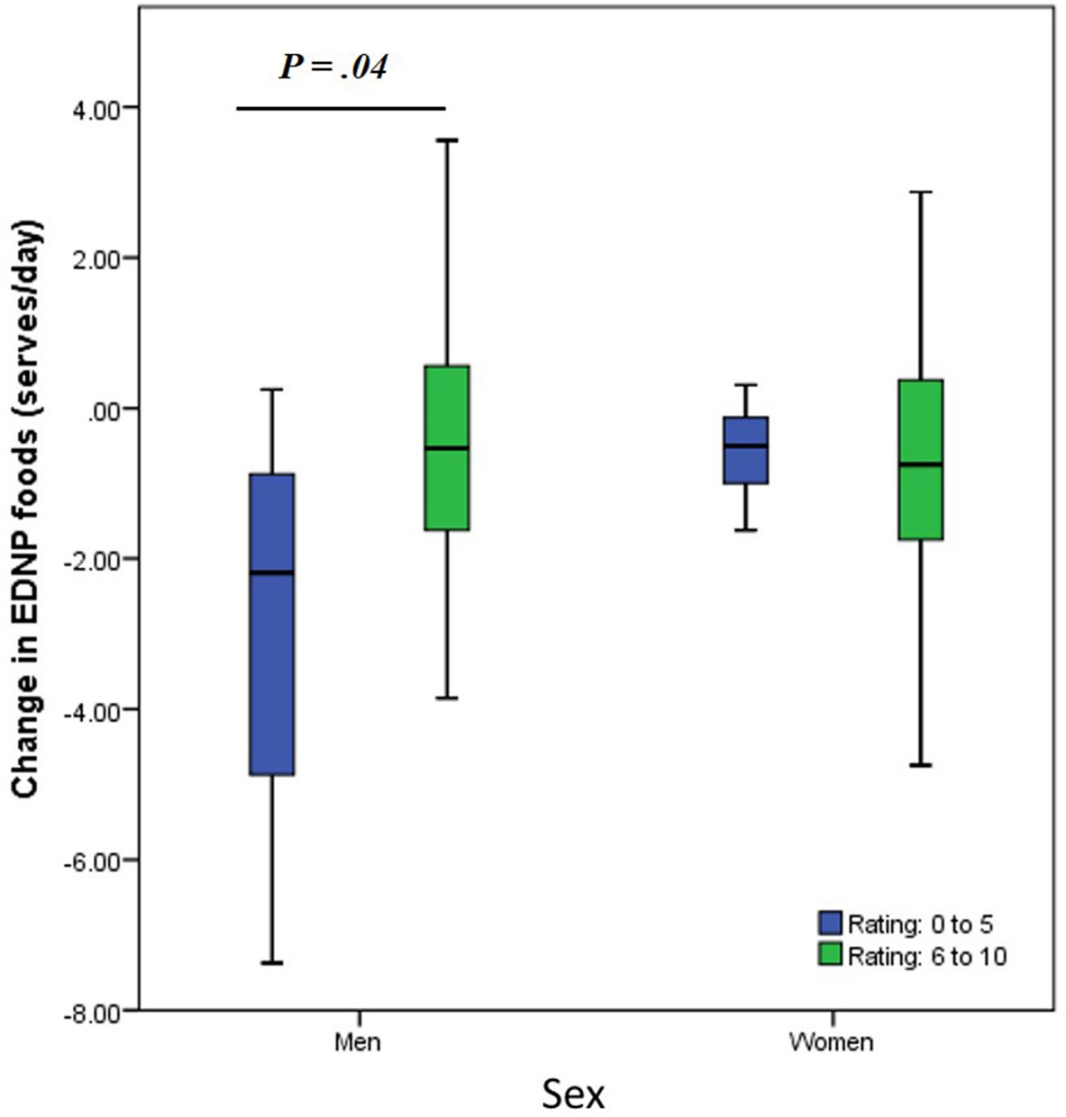
Interaction between sex and ‘importance of healthy eating’ on change in energy-dense nutrient poor (EDNP) food serves, for the two intervention arms of the study

There was a significant decrease in EDNP foods for men who received dietary feedback only compared to the control group (*P = .02*). Logistic regression analysis found men who received dietary feedback only were four times more likely to reduce their EDNP foods compared to controls (OR = 4.00 95 % CI [1.16–13.86]; *P = .03*). Participants were categorised into a high (6–10) and low (0–5) scores for the question ‘how important is eating a healthy diet’ (Fig. 4). Men in the two intervention arms who scored low on importance of healthy eating at baseline reduced their EDNP food serves per day significantly (*P = .04*) compared to men whose score was high.

Although not primary targets of the intervention, dietary feedback only group reduced weight *(P = .02)* and BMI *(P = .01)* significantly compared with the control group (Table 2). Further analysis of the dietary feedback only group showed the weight change from baseline was significant in those who were overweight (difference from baseline = −1.75 kg, 95 % CI = [−3.1, −0.4], *P = 0.01*), whereas no other BMI category (underweight, healthy weight or obese) reached statistical significance (p values ranging from 0.19 to 0.70). Control group participants who were in the healthy BMI category gained significant weight (difference from baseline =1.55 kg, 95 % CI = [0.57, 2.53], *P = .003*).

## Discussion

This 6-month randomized controlled trial showed using a mobile food record to inform tailored dietary feedback delivered via text messaging has promising potential for interventions targeting dietary intake and weight. Although the intervention was designed to be equally effective in both men and women this was not the case. Men who received tailored dietary feedback only showed a significant reduction of 1.4 serves of EDNP foods per day (equivalent to 840 kJ per day) compared with men in the control group. Men from both intervention groups reduced their intake of EDNP foods compared with baseline. For women, all groups increased their daily vegetable serves and reduced their EDNP food serves compared with baseline. Women in the intervention group receiving the dietary feedback only significantly reduced their daily intake of sugar-sweetened beverages, body weight and BMI compared with the control group. The participant retention was noticeably higher in the dietary feedback and text messaging (95 %) compared with the other groups (dietary feedback 88 % only and control 84 %), suggesting greater engagement may have occurred with this arm of the intervention.

The uniqueness of this study design includes the use of the mobile food record to collect food intake data and the use of text messaging to deliver tailored dietary feedback and nutrition messages. The analysis of the 4-day mFR formed the basis of the tailored dietary feedback text message. In demonstrating the usability of the mFR for this purpose we have addressed one of the criticisms of tailoring interventions, the lack of detailed dietary data for basing the feedback on [13]. Usually food frequency questionnaires or brief assessment methods are used as an outcome measurement instrument to assess fruit and vegetable intake, fibre or fat intake [31–34]. More detailed measures of diet such as food records are less often used due to concerns about respondent burden and costs associated with analysis. The high adherence rates achieved in this study show the mFR is a feasible dietary assessment method in young adults with potential for upscaling to larger population-based interventions.

The observed differences in intervention effects on fruits, vegetables, EDNP foods and SSB warrants further investigation, as do the changes from baseline. The fruits and vegetables text message provided tailored feedback on participants’ average daily serves intake compared with recommendations to eat two servings of fruit and five of vegetables each day. The Go for 2&5® high profile social marketing fruit and vegetable campaign has been conducted in Western Australia since 2001 and was running during the intervention period. The campaign targeted the main household food preparers (usually women) through television and other media and communications focused on increasing vegetable intake [35]. In the current study, women in both intervention arms and the control group increased their daily serves of vegetables above baseline values but men did not. The reason for this effect on women but not men may be due to either a dietary monitoring effect or influence of the Go for 2&5® campaign. The fruit and vegetable text messages (the feedback and the weekly texts) were based on the Go for 2&5® campaign messages. Different message content may be needed to motivate men in this age group to increase their vegetable intake [36].

In addition to the Go for 2&5® campaign, the LiveLighter® social marketing campaign which aims to encourage people to eat well, be physically active and maintain a healthy weight, commenced in Western Australia in June 2012 during the intervention period. Media advertisements encouraged limiting EDNP foods and beverages, particularly sugar-sweetened beverages (from July 2013). The tailored feedback on daily intake of serves of EDNP serves suggested changes they could make to their diet (see Fig. 3 for example message). Men in the dietary feedback only arm significantly reduced their EDNP foods whereas women in the dietary feedback only arm reduced their intake of SSB. These results may indicate the messages may have resonated with the target group and reinforced the campaign message. The focus groups that were conducted to inform the message development for this study found that messages to reduce EDNP food and SSB should incorporate both information and justification to be persuasive. There appeared to be low awareness of what constitutes EDNP foods and why they should be limited. At baseline, participants were consuming over four serves of EDNP food and beverages daily (equivalent to 2400 kJ) with EDNP making up around three serves. This is consistent with the findings of the recent Australian Health Survey which found EDNP food and beverages accounted for 35 % of average daily energy intake for young adults [5].

Reducing EDNP foods is an important public health intervention target and is consistent with the ‘small-change approach’ proposed by Hill [37] for addressing obesity at a population level. The intervention group receiving tailored dietary feedback significantly reduced their body weight by an average of 1.7 kg and BMI by 0.6 kg/m^2^ compared with the control group participants. Further, the weight reduction from baseline was significant in those who were overweight *(P = 0.01*). Although this intervention didn’t directly target body weight as a primary outcome, the results from this study suggest that tailored dietary feedback only appeared to have an important effect on reducing body weight in those who were overweight and that this change may have resulted from reduction in serves of EDNP foods.

Message tailoring appears to work by increasing the likelihood that people perceive the messages as personally relevant to them [24]. The tailoring used in this RCT was static and feedback was provided only once on the baseline assessment. It has been suggested that this type of tailoring is less effective than on-going dynamic tailoring [11]. The number of intervention contacts with participants is considered important in message tailoring [38]. However, there is limited evidence on the most effective aspects to guide text messaging interventions [39]. In the current study, one intervention arm, in addition to dietary feedback only also received weekly text messages designed to support and reinforce the dietary behaviours. The message was personalised with their name and participants could respond to the message. Although we would have ideally liked to have customized the weekly messages more, this was not feasible in the current study. The weekly text messages were more targeted communications rather than individually tailored [24]. A key finding from our focus group testing was the complexity of message development with no “one size fits all” [25]. Therefore we may not have framed the text messages in a way that was personally relevant to all participants. A priori, the hypothesis was the weekly text messages would be prompts for behaviour change. The additional intervention contacts did not appear to have any added benefit compared with dietary feedback alone. Perhaps the weekly text messaging dose was not adequate, however further research is needed to determine if this is the case.

To date there has been limited evaluation of nutrition text messaging interventions in healthy populations, with most focused on people with a chronic health condition such as diabetes or obesity [39, 40]. The weekly text messages were carefully constructed to be persuasive and increase motivation towards healthy eating behaviours [25]. However, the text messages may not have been perceived as personally relevant or more frequent and appropriate timing of messages may have had additional benefit. We were mindful of not burdening respondents or turning them off with too frequent interactions as there was little data in the literature on nutrition messages to guide the correct dose [41]. We relied on focus group advice prior to the intervention to set the weekly message dose. Only two participants in the intervention group receiving the weekly text messages opted to stop receiving them suggesting that message fatigue was not an issue. One cannot rule out that participants may have opted out by ignoring the messages or simply deleted them rather than choosing to formally stop the messages. From these results, further research is needed to identify the factors associated with text messaging acceptance, including message content for specific dietary behaviours, in healthy young adults.

A major strength of this study was the high retention level achieved which may be partly attributed to the level of engagement in technology by using the mFR App. Although the control group only had two interactions with the research team six months apart, 89 % completed the study. In our previous studies we have emphasised the importance of obtaining user feedback [42]. The request for usability feedback on the novel CHAT App at baseline and 6-month may have contributed to better engagement than is typically observed with other dietary assessment methods. A criticism of the design of technology-based behavioural interventions is the lack of behaviour change models to inform them [43, 44]. Mohr et al. [43] proposed a Behavioural Intervention Technology (BIT) framework for interventions using a range of technologies, including mobile telephones, the internet and sensors. Features such as usability and willingness to continue to use the App may contribute to greater engagement and motivation enhancement by participants [43]. The current intervention was designed on theoretical constructs from SDT and MI [18–20] but future technology-based interventions may need to consider other novel constructs that take these features into account. The text message content was developed to support autonomous decision making and the ‘tone of voice’ and language used in all communications was consistent with SDT. The intervention also drew on the researchers formative focus group findings which found that providing practical solutions to barriers to healthy eating important, as well as including access to healthy, cheap, quick and easy to prepare recipes adapted to a mobile phone platform [25].

### Limitations

Although we attempted to recruit a population-based sample by using the electoral role the responders may not have been representative of the population. The response rate from women was higher than men. This is consistent with other population studies in Western Australia that have found it is more difficult to recruit men into studies than women [45]. However, the participants recruited were from a diverse background for socio-economic status and ethnicity.

We selected young adults as these are a group in transition from adolescents to adulthood and where improving dietary habits and preventing weight gain is important for the prevention of chronic diseases. Text messaging interventions in health have wide appeal to public health researchers as there is direct delivery of the message to participants. The mobile telephone is increasingly used to send reminders to people about appointments. Therefore people may ‘turn off’ to text messages not perceived as directly relevant to them. In the current study, intervention group participants received two personalised dietary feedback messages delivered as text messages, related to their fruit and vegetable and junk food intake. Although positive effects were observed, it is possible that with a higher dose (more frequent dietary feedback), additional changes may have been observed. Long periods of dietary monitoring may also improve outcomes. The current mFR has been designed more as an assessment tool rather than a self-monitoring tool. However, in the future, the mFR could be modified for the dual purpose of assessment and self-monitoring of diet.

The lack of effect observed with the weekly messages also requires further exploration. Our hypothesis was that the more intensive intervention with greater contact points would be more effective but this was not the case. The weekly text messages, designed to support behaviour change, were personalised with the name of the person but it is possible the content of the message may not have been relevant or sent at an appropriate time. The content of the weekly messages had been constructed from focus group work prior to the intervention [25]. As found in other research in overweight and obese adolescents [41, 46], what people say they want in a text message versus their actual experience in receiving the text message may not be the same. Process evaluation of the text messages may assist in exploring these issues to inform future interventions. A further limitation was that there was no follow up after the 6-month intervention period to evaluate if the observed changes were maintained over time.

Misreporting of dietary intake is common to most dietary assessment methods and cannot be ruled out in the current study [47]. Misreporting of intake may have occurred due to participants either not recording all food and beverages consumed or modifying their usual intake during the record period. Reactivity bias may have occurred with the mobile food record; however the control group also undertook the mFR recording (same time points and length of recording) but did not receive feedback. We would expect the reactivity bias to be similar across the groups. The findings presented here are based on food group servings of fruit, vegetables and EDNP foods and beverages, rather than grams and nutrients. A possible limitation of the study was that the manual assessment of food group serves by a trained analyst may not be sensitive enough to detect small but meaningful changes in dietary habits, for example 0.25 serve increase in fruit or vegetables. Food recording whether by paper, digital entry or image-based requires an estimation of portion size by either the participant or the researcher. For the mFR the participant was not required to record the portion size consumed. The trained analyst, used the fiducial marker (a scaling device) in the image to assist with portion size estimation. In the current study, as the dietary analysis was undertaken by a trained analyst the resource implications of the mFR were not fully explored. The inclusion of an economic evaluation of the trained analyst would be an important inclusion for future studies using the mFR to evaluate the potential for upscaling in larger populations. Future planned improvements to automate the image analysis for the mobile food record may improve the accuracy of the dietary assessment [16]. In addition, further analysis of post-intervention feedback on the text messages may guide future improvements in the methodology.

## Conclusions

This 6-month RCT has demonstrated the potential of the image-based mobile food record as a feasible method for collecting dietary data in young adults. In addition, we have been able to show the importance of dietary feedback in promoting behaviour change. The effect of the dietary feedback intervention on reduction in body weight was an unexpected finding and requires further investigation to confirm these results. This innovative approach making best use of technology for the collection of dietary data and delivering tailored feedback direct to the individual may provide an efficient delivery method for health promotion programs that target this hard to reach population group.

## References

[1] Malik VS, Pan A, Willett WC, Hu FB (2013). Sugar-sweetened beverages and weight gain in children and adults: a systematic review and meta-analysis. Am J Clin Nutr.

[2] World Health Organisation (2011). Global status report on noncommunicable diseases 2010.

[3] Australian Bureau of Statistics (2013). Profiles of Health, Australia, 2011-13, cat. no. 4338.0.

[4] Swinburn B, Kraak V, Rutter H, Vandevijvere S, Lobstein T, Sacks G (2015). Strengthening of accountability systems to create healthy food environments and reduce global obesity. Lancet.

[5] Australian Bureau of Statistics (2013). Australian Health Survey: Nutrition First Results - Foods and Nutrients, 2011-12, cat. no. 4364.0.55.007.

[6] Hebden L, Chey T, Allman-Farinelli M (2012). Lifestyle intervention for preventing weight gain in young adults: a systematic review and meta-analysis of RCTs. Obes Rev.

[7] Burke LE, Wang J, Sevick MA (2011). Self-monitoring in weight loss: a systematic review of the literature. J Am Diet Assoc.

[8] Rosner B, Gore R (2001). Measurement error correction in nutritional epidemiology based on individual foods, with application to the relation of diet to breast cancer. Am J Epidemiol.

[9] Boushey CJ, Kerr DA, Wright J, Lutes KD, Ebert DS, Delp EJ (2009). Use of technology in children’s dietary assessment. Eur J Clin Nutr.

[10] Kreuter MW, Skinner CS (2000). Tailoring: what’s in a name?. Health Educ Res.

[11] Krebs P, Prochaska JO, Rossi JS (2010). A meta-analysis of computer-tailored interventions for health behavior change. Prev Med.

[12] Kroeze W, Werkman A, Brug J (2006). A systematic review of randomized trials on the effectiveness of computer-tailored education on physical activity and dietary behaviors. Ann Behav Med.

[13] Broekhuizen K, Kroeze W, van Poppel MN, Oenema A, Brug J (2012). A systematic review of randomized controlled trials on the effectiveness of computer-tailored physical activity and dietary behavior promotion programs: an update. Ann Behav Med.

[14] Kirkpatrick SI, Reedy J, Butler EN, Dodd KW, Subar AF, Thompson FE (2014). Dietary assessment in food environment research: a systematic review. Am J Prev Med.

[15] Bosch M, Zhu F, Khanna N, Boushey CJ, Delp EJ (2011). Combining global and local features for food identification in dietary assessment. IEEE Trans Image Process.

[16] Zhu F, Bosch M, Khanna N, Boushey CJ, Delp EJ (2015). Multiple hypotheses image segmentation and classification with application to dietary assessment. IEEE J Biomedical Health Informatics.

[17] Kerr DA, Pollard CM, Howat P, Delp EJ, Pickering M, Kerr KR (2012). Connecting Health and Technology (CHAT): protocol of a randomized controlled trial to improve nutrition behaviours using mobile devices and tailored text messaging in young adults. BMC Public Health.

[18] Vansteenkiste M, Sheldon KM (2006). There’s nothing more practical than a good theory: integrating motivational interviewing and self-determination theory. Br J Clin Psychol.

[19] Deci EL, Ryan RM (2012). Self-determination theory in health care and its relations to motivational interviewing: a few comments. Int J Behav Nutr Phys Act.

[20] Patrick H, Williams GC (2012). Self-determination theory: its application to health behavior and complementarity with motivational interviewing. Int J Behav Nutr Phys Act.

[21] Australian Bureau of Statistics (2006). Census of Population and Housing: Socio-Economic Indexes for Areas (SEIFA), Australia - Data only 2006 (cat. no. 2033.0.55.001) and Information Paper: An Introduction to Socio-Economic Indexes for Areas (SEIFA), 2006 (cat. no. 2039.0).

[22] Schap TE, Boushey CJ (2011). Reported energy intake among adults using the mobile telephone food record does not differ from estimated energy requirements. FASEB J.

[23] Smith A, Kellett E, Schmerlaib Y (1998). The Australian Guide to Healthy Eating: Background Information for Nutrition Educators. Commonwealth Department of Health & Family Services, ed.

[24] Hawkins RP, Kreuter M, Resnicow K, Fishbein M, Dijkstra A (2008). Understanding tailoring in communicating about health. Health Educ Res.

[25] Pollard CM, Howat PA, Pratt IS, Boushey CJ, Delp EJ, Kerr DA (2016). Preferred tone of nutrition text messages for young adults: focus group testing. JMIR mHealth uHealth.

[26] Resnicow K, Davis RE, Zhang G, Konkel J, Strecher VJ, Shaikh AR (2008). Tailoring a fruit and vegetable intervention on novel motivational constructs: results of a randomized study. Ann Behav Med.

[27] Pollard CM, Nicolson C, Pulker CE, Binns CW (2009). Translating government policy into recipes for success! Nutrition criteria promoting fruits and vegetables. J Nutr Educ Behav.

[28] Stewart A, Marfell-Jones MJ, Olds TS, de Ridder H (2011). International standards for anthropometric assessment.

[29] Daly A, Pollard CM, Kerr DA, Binns CW, Phillips M (2015). Using short dietary questions to develop indicators of dietary behaviour for use in surveys exploring attitudinal and/or behavioural aspects of dietary choices. Nutrients.

[30] Craig CL, Marshall AL, Sjostrom M, Bauman AE, Booth ML, Ainsworth BE (2003). International physical activity questionnaire: 12-country reliability and validity. Med Sci Sports Exerc.

[31] Smeets T, Kremers SP, Brug J, de Vries H (2007). Effects of tailored feedback on multiple health behaviors. Ann Behav Med.

[32] Gans KM, Risica PM, Strolla LO, Fournier L, Kirtania U, Upegui D (2009). Effectiveness of different methods for delivering tailored nutrition education to low income, ethnically diverse adults. Int J Behav Nutr Phys Act.

[33] Wright JL, Sherriff JL, Dhaliwal SS, Mamo JC (2011). Tailored, iterative, printed dietary feedback is as effective as group education in improving dietary behaviours: results from a randomised control trial in middle-aged adults with cardiovascular risk factors. Int J Behav Nutr Phys Act.

[34] Fries E, Edinboro P, McClish D, Manion L, Bowen D, Beresford SA (2005). Randomized trial of a low-intensity dietary intervention in rural residents: the rural physician cancer prevention project. Am J Prev Med.

[35] Pollard C, Daly AM, Binns CW (2009). Consumer perceptions of fruit and vegetables serving sizes. Public Health Nutr.

[36] Glasson C, Chapman K, James E (2011). Fruit and vegetables should be targeted separately in health promotion programmes: differences in consumption levels, barriers, knowledge and stages of readiness for change. Public Health Nutr.

[37] Hill JO (2009). Can a small-changes approach help address the obesity epidemic? A report of the Joint Task Force of the American Society for Nutrition, Institute of Food Technologists, and International Food Information Council. Am J Clin Nutr.

[38] Noar SM, Benac CN, Harris MS (2007). Does tailoring matter? Meta-analytic review of tailored print health behavior change interventions. Psychol Bull.

[39] Hall AK, Cole-Lewis H, Bernhardt JM (2015). Mobile text messaging for health: a systematic review of reviews. Annu Rev Public Health.

[40] Siopis G, Chey T, Allman-Farinelli M (2015). A systematic review and meta-analysis of interventions for weight management using text messaging. J Hum Nutr Diet.

[41] Smith KL, Kerr DA, Fenner AA, Straker LM (2014). Adolescents just do not know what they want: a qualitative study to describe obese adolescents’ experiences of text messaging to support behavior change maintenance post intervention. J Med Internet Res.

[42] Boushey CJ, Harray AJ, Kerr DA, Schap TE, Paterson S, Aflague T (2015). How willing are adolescents to record their dietary intake? The mobile food record. JMIR mHealth uHealth.

[43] Mohr DC, Schueller SM, Montague E, Burns MN, Rashidi P (2014). The behavioral intervention technology model: an integrated conceptual and technological framework for eHealth and mHealth interventions. J Med Internet Res.

[44] Riley WT, Rivera DE, Atienza AA, Nilsen W, Allison SM, Mermelstein R (2011). Health behavior models in the age of mobile interventions: are our theories up to the task?. Trans Behav Med.

[45] Pollard CM, Daly A, Moore M, Binns CW (2013). Public say food regulatory policies to improve health in Western Australia are important: population survey results. Aust N Z J Public Health.

[46] Woolford SJ, Barr KL, Derry HA, Jepson CM, Clark SJ, Strecher VJ (2011). OMG do not say LOL: obese adolescents’ perspectives on the content of text messages to enhance weight loss efforts. Obesity (Silver Spring).

[47] Subar AF, Freedman LS, Tooze JA, Kirkpatrick SI, Boushey C, Neuhouser ML (2015). Addressing Current Criticism Regarding the Value of Self-Report Dietary Data. J Nutr..

